# Association of gait with global cognitive function and cognitive domains detected by MoCA-J among community-dwelling older adults: a cross-sectional study

**DOI:** 10.1186/s12877-021-02467-5

**Published:** 2021-10-02

**Authors:** Wen Hao, Wenjing Zhao, Takashi Kimura, Shigekazu Ukawa, Ken Kadoya, Katsunori Kondo, Akiko Tamakoshi

**Affiliations:** 1 Department of Public Health, Hokkaido University Graduate School of Medicine, Sapporo, Hokkaido, Japan; 2 Research Unit of Advanced Interdisciplinary Care Science, Graduate School of Human Life Science, Osaka City University, Osaka, Japan; 3 Department of Orthopedic Surgery, Hokkaido University Graduate School of Medicine, Sapporo, Hokkaido Japan; 4 Department of Social Preventive Medical Sciences, Center for Preventive Medical Sciences, Chiba University, Chiba, Japan; 5 Department of Gerontological Evaluation, Center for Gerontology and Social Science, National Center for Geriatrics and Gerontology, Obu City, Aichi Japan

**Keywords:** Aged, Dementia, Gait, Cognition, Executive function, Factor analysis

## Abstract

**Background:**

Gait was proved to be strongly associated with global cognitive function and multiple cognitive domains; however, previous research usually concentrated on individual gait parameters. This study used wearable sensors to measure gait parameters in different aspects and comprehensively explored the association of gait with global cognitive function and cognitive domains.

**Methods:**

The data of this cross-sectional study were obtained from 236 community-dwelling Japanese older adults (125 men and 111 women) aged 70–81 years. Gait was measured by asking participants to walk a 6-m course and back using the Physilog® sensors (GaiUp®, Switzerland). Global cognitive function and cognitive domains were evaluated by face-to-face interviews using the Japanese version of the Montreal Cognitive Assessment. Twenty gait parameters were summarized as independent gait factors using factor analysis. A generalized linear model and linear regression model were used to explore the relationship of gait with global cognitive function and cognitive domains adjusted for several confounding factors.

**Results:**

Factor analysis yielded four gait factors: general cycle, initial contact, propulsion, and mid-swing. Among them, general cycle factor was significantly associated with global cognitive function (β = − 0.487, [− 0.890, − 0.085]) and executive function (*P* = 0.049); initial contact was associated with executive function (*P* = 0.017).

**Conclusion:**

General cycle of gait might be the better marker of global cognitive function and gait is most strongly associated with executive function. The longitudinal relationships should be examined in future cohort studies.

**Supplementary Information:**

The online version contains supplementary material available at 10.1186/s12877-021-02467-5.

## Background

Gait is the most important method of human locomotion, characterized by periods of loading and unloading of the limbs to move around and provide independence [1]. Usually, gait incorporates several measurable gait parameters, such as speed, stride length, stride width, and cadence. Emerging epidemiological studies, have reported strong relationship of gait parameters with both global cognitive function and cognitive domains among older adults. Preferred gait speed, as one of the most frequently studied parameters, was proved to be related with global cognitive dysfunction in community-dwelling older adults [2] and older users of primary care service [3]. Slower gait speed was also indicated to be related with worse executive function, immediate memory [4], and worse attention [5] in cognitively healthy older adults. In a Japanese study, maximum gait speed was claimed to be better correlated with global cognitive function than normal gait speed [6]. Cadence was related with executive function and memory; stride length was related with global cognitive function [4].

Nevertheless, gait is multidimensional and cannot be evaluated by a single characteristic. The individual parameter applied before could not show a comprehensive association between gait and cognitive function. Recently, a wearable sensor has been invented that can measure gait from general, temporal, visual, and clearance aspects, while simultaneously allowing people to walk in a natural environment. Moreover, based on the knowledge that quantitative gait parameters are highly correlated with each other and their individual relationships with cognitive function may be difficult to observe while adjusting for other gait variables [7], conceptual models using principal component analysis or factor analysis to provide a simplified framework for selecting grouped gait factors were suggested in gait analysis [8]. Under these circumstances, research that includes gait parameters from different aspects and uses a conceptual model for systemically analyzing gait and cognitive function is scarce.

Therefore, in the current study, we adopted a more advanced sensor that can obtain 20 gait parameters and expected that grouped gait factors extracted by conceptual models would provide a new insight into gait analysis. Meanwhile, we aimed to examine the associations of gait with both global cognitive function and cognitive domains among community-dwelling older adults. It is hypothesized that not only temporal factor but all the aspects of gait will be found to be related with global cognitive function and cognitive domains.

## Methods

### Participants

The data of this research were obtained from the Cognition and Activity in Rural Environment of HokkaiDO Senior (CARE-DO) summer survey 2018. The CARE-DO study is a prospective cohort study embedded in the Japan Gerontological Evaluation Study (Jages) 2016.The detailed information about Jages has been described elsewhere [9]. Briefly, it is a large panel study directed at understanding the health, social, and behavioral issues among the older population in Japan. The Jages 2016 wave included more than 200,000 citizens aged 65 and above and who did not have long-term care insurance from 39 cities or towns in Japan. From this pool, the baseline study of CARE-DO invited those who lived in six towns of Hokkaido and aged 69–80 to the CARE-DO winter survey 2017 to understand the influence of indoor temperature distribution on health of the elderly in cold climate [10]. In the second year, we invited all 569 participants who had responded to the winter survey 2017 by post card to attend the current CARE-DO summer survey 2018 conducted at September 2018 to explore associations between gait and cognition in older adults. Until the beginning of the investigation, 260 people did not respond and two asked their partners to come instead. Due to the small sample size and the aim of exploring cross-sectional relationships, their participation was also recognized. Therefore, a total of 309 people were regarded as the participants of this study.

**Fig. 1:**
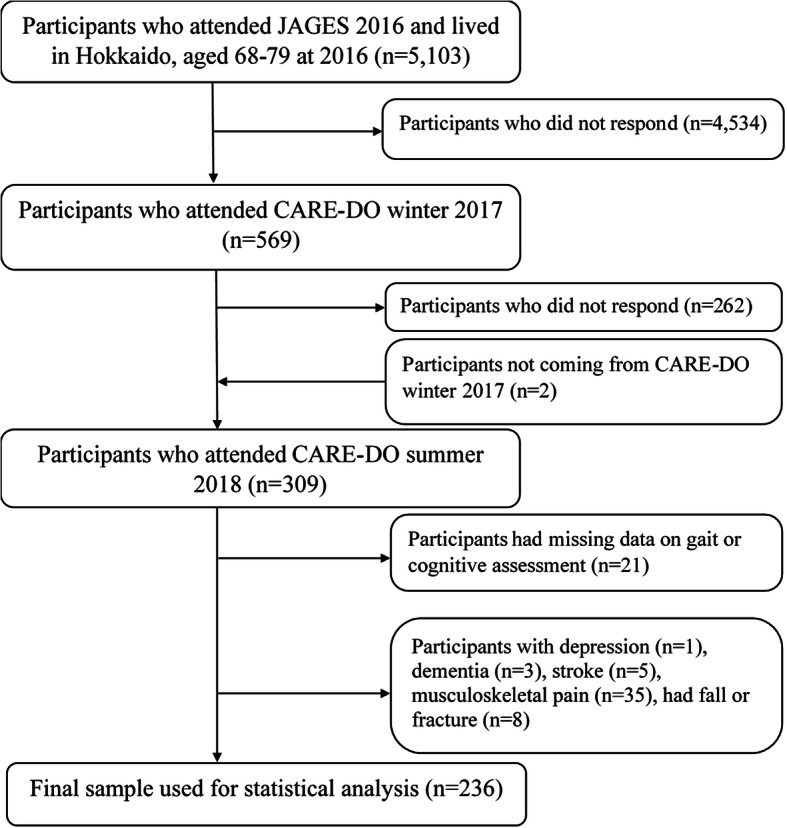
Flowchart of study participants. JAGES, the Japan Gerontological Evaluation Study; CARE-DO, Cognition and Activity in Rural Environment of HokkaiDO senior

The CARE-DO summer survey 2018 included four main parts: physical examinations, gait and cognitive assessments, and a self-administered questionnaire. Height and weight were measured using the corresponding scale during the physical examination. Body mass index was calculated as weight in kilogram divided by height in meters squared. After the participants completed all assessments, a self-administered questionnaire that contained information on age, sex, functional activity, and history of diseases, such as diabetes (yes or no) and hypertension (yes or no), were allocated to each of them and expected to be sent back after 2 weeks. The functional activity was evaluated as instrumental activities of daily living (IADL) score using subtest of the Tokyo Metropolitan Institute of Gerontology Index of Competence, which is a validated 13-item self-reported index and higher score indicated better functional capacity [11]. After excluding 21 participants with missing data on cognitive and gait assessments; one with depression; three with Parkinson’s disease or Alzheimer’s disease; five with stroke; 35 with musculoskeletal pain; and eight who once fell down or had a bone fracture; 236 participants were regarded as valid participants and were included in the data analysis process (Fig. 1).

This study was approved by the ethics committee of the Graduate School of Medicine, Hokkaido University (no.18–025), all the experiment protocol for involving human data was in accordance with the relevant guidelines. A written informed consent was obtained from all the participants.

### Gait measurement

All participants were asked to walk a round trip of a 6-m straight path at a self-selected walking speed. Gait was measured using Physilog® sensors (GaitUp®, Switzerland), which consist of two small, lightweight (19 g), inertial sensors for each foot, and elastic straps to attach the sensors to the dorsum of the foot, and Gait Analyzer software version 3.1 (GaitUp) running on a Windows personal computer. Two sensors on both the feet could be synchronized wirelessly, and no calibration procedure was required before and during the measurement. The position of the sensor on the foot did not affect the measurements [12]. The following 21 kinematic gait parameters from four aspects could be obtained through this device: general (cycle duration, cadence, stride time, stride velocity, and turning angle); temporal (stance, swing, loading, foot-flat, pushing, and double support); spatial (peak angle velocity, swing speed, strike angle, lift-off angle, swing width, and three-dimensional (3D) path length); and clearance (maximum heel clearance, maximum toe clearance 1, minimum toe clearance, and max toe clearance 2). The validity of this gait assessment has been published elsewhere [13]. As gait cycle duration is just a different format of cadence, the parameter of gait cycle duration was excluded before the analysis. The data at the time of accelerating or decelerating and making a turn were excluded by the default function of the Gait Analyzer. The mean values of two feet were used. Detailed descriptions of these parameters are provided in Additional file 1.

### Cognition measurement

Global cognitive function was evaluated using the Japanese version of the Montreal Cognitive Assessment (MoCA-J) through face-to-face interviews. The MoCA-J is a one-page 30-point test and has been reported to have good internal consistency reliability (Cronbach’s alpha = 0.74), mild cognitive impairment (MCI) was defined as MoCA-J’s score less than 26 [14]. The total evaluating time was around 15 min, and higher scores represent better cognitive function. To regulate the effect of education, participants with an educational background of less than 12 years were asked to add one score to the total score. Internal subtests of MoCA-J were used to evaluate the following six cognitive domains: memory (five points): delayed recall; executive function (four points): letter fluency, trial making, verbal abstraction; visuospatial (four points): cube copy, clock drawing; languages (five points): naming, sentence repetition; attention (six points): digit span forward and backward, letter A tapping, serial-7 subtraction; and orientation (six points): orientation of date and place [15].

### Statistical analysis

The chi-square test was used to compare the categorical variables, and a one-way analysis of variance was used to compare the continuous variables.

All 20 continuous gait parameters were standardized to have a mean of 0 and a standard deviation of one because of the different scales before analysis. Subsequently, factor analysis using the principal component method was performed to reduce the larger number of highly correlated variables to a smaller number of uncorrelated independent predictors. The initial factors were subjected to an orthogonal quartimax rotation. Gait parameters with a rotated loading of > 0.5 were considered as the dominant contributors to the main factors. The name was labeled according to the interpretation of the original gait parameters.

A generalized linear model was used to identify the relationship between the MoCA-J score and retained gait factors in all the participants and both sexes. Due to the total score of each cognitive subtest was small and direct adoption of the linear regression model may cause bias, tertile was made for each gait factor. The mean value of each group was adjusted for age, sex, height, and education as the least square mean value. *P* for trends were tested by linear regression model, in all the participants and both sexes, test for trend based on variable containing median value for each tertile. The main confounding factors in this study were age, sex, height, weight, educational status, diabetes, hypertension, and IADL scores. Statistical significance was set at *P* < 0.05. All statistical analyses were performed using the SAS software (version 9.4; SAS Institute Inc., Cary, NC, USA).

## Results

The study included 125 men and 111 women with an average age of 75.28 years and 75.53 years, respectively. Approximately 45% of the patients had hypertension, 14% had diabetes and 72% had MCI. Men had significantly higher weight, taller height and lower IADL scores than women. In gait assessments, women had significantly shorter stride length, quicker cadence, and shorter stance period than men. In cognitive measurements, women performed significantly better on subtest of memory (Table 1).

**Table 1:** Characteristics of participants according to sex

Age (years)		Men (*n* = 125)		Women (*n* = 111)		*P*
		75.28	(2.84)	75.53	(2.81)	0.658^†^
Height (m)	mean (SD)	162.99	(5.08)	150.63	(5.22)	**< 0.001**^†^
Weight (kg)	mean (SD)	62.90	(8.09)	52.35	(8.92)	**< 0.001**^†^
BMI (kg/m^2^)	mean (SD)	23.67	(2.73)	23.06	(3,06)	**0.158**^†^
12 years of education	n (%)	35	(26)	16	(14)	**0.014**^‡^
IADL score	mean (SD)	12.16	(1.21)	12.41	(0.98)	**0.023**^†^
Gait measurements						
stride length (m)	mean (SD)	1.08	(0.12)	1.02	(0.14)	**0.005**^†^
speed (m/s)	mean (SD)	0.98	(0.16)	1.00	(0.17)	0.274^†^
cadence (steps/min)	mean (SD)	110.47	(8.89)	117.91	(11.81)	**< 0.001**^†^
stance (s)	mean (SD)	0.68	(0.07)	0.65	(0.09)	**< 0.001**^†^
History of diseases						
Hypertension	n (%)	61	(45)	46	(41)	0.433^‡^
Diabetes	n (%)	21	(16)	11	(10)	0.136^‡^
Cognitive measurements						
MCI	n (%)	96	(71.1)	75	(67.6)	0.144^‡^
MoCA-J total score (points)	mean (SD)	23.08	(3.26)	23.68	(3.12)	0.476^†^
Subtest’s score (points)						
executive (0–4)	mean (SD)	2.74	(0.99)	2.73	(1.03)	0.962^†^
language (0–5)	mean (SD)	3.82	(0.79)	3.70	(0.73)	0.293^†^
memory (0–5)	mean (SD)	2.43	(1.71)	3.13	(1.66)	**< 0.001**^†^
orientation (0–6)	mean (SD)	5.70	(0.58)	5.77	(0.62)	0.307^†^
visuospatial (0–4)	mean (SD)	2.61	(0.71)	2.62	(0.7)	0.707^†^
attention (0–6)	mean (SD)	5.05	(1.15)	4.88	(1.06)	0.275^†^

Bolded *P*-values indicate *P* < 0.05. BMI, body mass index. IADL, instrumental activities of daily living. MCI, mild cognitive impairment. MoCA-J, Japanese version of Montreal Cognitive Assessment; SD, standard deviation. ^†^one-way analysis of variance, ^‡^chi-square test

**Fig. 2:**
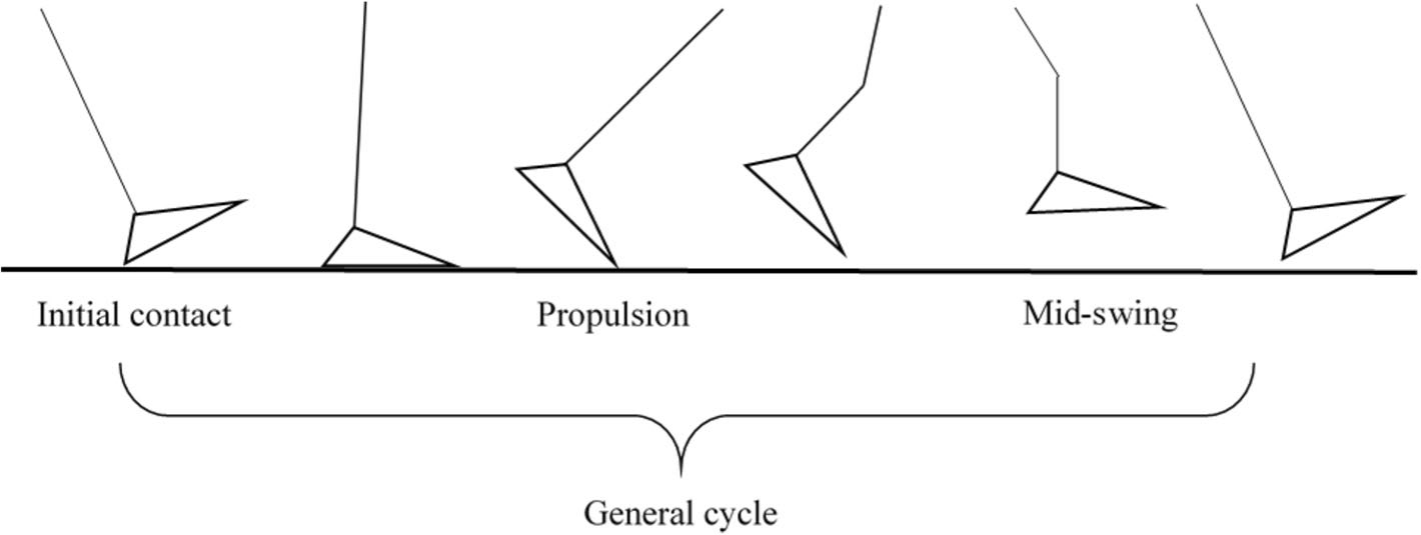
Four independent gait factors extracted by factor analysis

Factor analysis with quartimax rotation yielded exactly four orthogonal factors that accounted for 89.17% of the variance in 20 gait parameters (Table 2). The factor with the highest variance had strong loadings on stance, cadence, foot-flat, stride velocity, double support, swing, swing speed and peak angle velocity and was termed “general cycle” factor. The second factor reflecting the strike angle, maximum toe clearance stride length loading, and 3D path length was named “initial contact” factor. The third factor loaded on pushing and lift-off angle was termed “propulsion” factor. The final factor loaded heavily on maximum heel clearance and maximum toe clearance and was called “mid-swing” factor (Fig. 2). A lower score for the general cycle factor and propulsion factor represented better gait, and a higher score of initial contact and mid-swing factor represented better gait.

**Table 2:** Factor loadings of 20 gait variables on four independent gait factors extracted by factor analysis

Gait variable	General cycle	Initial contact	Propulsion	Mid-swing
Stance (s)	0.97			
Cadence (steps/min)	−0.95			
Foot-flat (s)	0.90			
Stride velocity (m/s)	−0.82			
Double support (s)	0.80			
Swing (s)	0.75			
Swing speed (m/s)	−0.75			
Peak angle velocity (degree/s)	−0.74			
Strike angle (degree)		0.91		
Stride length (m)		0.83		
Maximum toe clearance 2 (m)		0.83		
3D path length (meters)		0.79		
Loading (s)		0.69		
Stride width (m)		0.38		
Turning angle (degree)		0.29		
Pushing (s)			0.89	
Lift-off angle (degree)			0.68	
Minimum toe clearance (m)			−0.47	
Maximum heel clearance (m)				0.84
Maximum toe clearance 1(m)				0.83
Variance explained (%)	43.27	24.56	13.31	8.03

### Gait and global cognitive function

Of the four gait factors, general cycle and propulsion factor had a negative association with the MoCA-J score, and initial contact and mid-swing factor had a positive association with MoCA-J score. The general cycle factor showed significant associations with global cognitive function in the crude model (β = − 0.674 [− 1.074, − 0.274]); model adjusted for age, sex, height, and weight (β = − 0.562, [− 0.964, − 0.160]); and model adjusted for age, sex, height, weight diabetes, hypertension, and IADL scores (β = − 0.487, [− 0.890, − 0.085]) of all the participants. The factor of the general cycle was also significantly associated with global cognition in the crude model (β = − 0.827 [− 1.357, − 0.296]); model adjusted for age, height, and weight (β = − 0.773, [− 1.300, − 0.247]); and model adjusted for age, height, weight, education, diabetes, hypertension, and IADL scores (β = − 0.651, [− 1.191, − 0.110]). In men, significance was found neither in the crude model nor in the adjusted models (Table 3).

**Table 3:** Associations between gait factors and global cognitive function in all participants and both sexes

Model	Total			Women			Men		
	β	Wald 95% Confidence limits		β	Wald 95% Confidence limits		β	Wald 95% Confidence limits	
Model 1^†^									
General cycle	−0.674***	−1.074	−0.274	−0.827**	−1.357	− 0.296	−0.403	−1.046	0.240
Initial contact	0.232	−0.180	0.645	0.343	−0.269	0.955	0.328	−0.260	0.916
Propulsion	−0.038	−0.453	0.376	−0.343	− 0.949	0.264	0.011	−0.623	0.644
Mid-swing	0.145	−0.286	0.575	0.388	−0.403	1.180	0.220	−0.356	0.795
Model 2^‡^									
General cycle	−0.562**	−0.964	− 0.160	−0.773**	−1.300	− 0.247	−0.361	− 0.980	0.257
Initial contact	0.114	−0.301	0.534	0.190	−0.435	0.815	0.074	−0.489	0.637
Propulsion	−0.164	−0.591	0.263	−0.370	− 0.977	0.237	0.044	−0.550	0.638
Mid-swing	0.082	−0.372	0.536	0.304	−0.508	1.117	−0.066	−0.611	0.480
Model 3^§^									
General cycle	−0.487*	−0.890	− 0.085	−0.651*	−1.191	− 0.110	−0.360	− 0.974	0.254
Initial contact	0.086	−0.335	0.507	0.131	−0.767	0.504	0.199	−0.381	0.779
Propulsion	−0.257	−0.677	0.164	−0.421	−1.007	0.166	−0.113	− 0.720	0.493
Mid-swing	−0.074	−0.527	0.380	0.057	−0.758	0.871	−0.184	−0.725	0.356

MoCA-J, Japanese version of Montreal Cognitive Assessment

^†^Model 1 crude model

^‡^Model 2 adjusted for age, sex, weight, and height

^§^Model 3 adjusted for age, sex, weight, height, diabetes, hypertension, and IADL scores

*P < 0.05, **P < 0.01, ***P < 0.001

### Gait and cognitive domains detected by MoCA-J

Of the six cognitive domains, significant differences were found in the general cycle factor (*P* = 0.049), initial contact factor (*P* = 0.017) with executive domain. Better performance of the initial contact was correlated with higher scores of executive function (Table 4). Men and women showed different results. Better performance of initial contact was related to higher scores of executive function and better performance of mid-swing was related with better orientation function in women (Additional Table 2); no significant relationship was found between gait and specific domain in men (Additional Table 3).

**Table 4:** Adjusted means of internal subtests by tertile of each gait factor in all the participants

Factor		Executive function	Language	Memory	Orientation	Visuospatial	Attention
General cycle	Low	2.91	3.70	2.93	5.75	2.58	5.09
	Moderate	2.76	3.79	2.78	5.80	2.69	4.93
	High	2.54	3.81	2.57	5.65	2.57	4.89
*P* for trend		**0.049**	0.362	0.231	0.283	0.697	0.424
Initial contact	Low	2.53	3.84	2.74	5.74	2.58	4.96
	Moderate	2.72	3.76	2.77	5.71	2.64	4.92
	High	2.96	3.70	2.77	5.75	2.63	5.03
*P* for trend		**0.017**	0.275	0.857	0.942	0.850	0.587
Propulsion	Low	2.80	3.68	2.82	5.70	2.61	5.07
	Moderate	2.66	3.91	2.60	5.83	2.64	4.91
	High	2.75	3.71	2.85	5.67	2.60	4.94
*P* for trend		0.557	0.809	0.981	0.720	0.741	0.450
Mid-swing	Low	2.56	3.70	2.87	5.61	2.54	4.91
	Moderate	2.85	3.86	2.64	5.79	2.74	5.03
	High	2.79	3.73	2.76	5.79	2.56	4.97
*P* for trend		0.322	0.889	0.693	0.086	0.555	0.738

All scores are displayed as least square mean values adjusted for age, sex, education, and height; *P* for trends were tested by linear regression models adjusted for age, sex, education, height, weight, diabetes, hypertension, and IADL score

## Discussion

Our study found that among older adults aged 70–81 years, the general cycle is most associated with global cognitive function; with respect to cognitive domains detected by MoCA-J, results showed better general cycle and initial contact phase related to better executive function.

To the best of our knowledge, this is the first study to include most of the various gait parameters that almost consist of the whole gait phase when studying the association between gait and cognitive function. To date, various techniques have been used in gait analysis, including stopwatches, electronic walkways, body-worn sensors, electromyography, and 3D motion analysis [1, 8]. The technique used in this study was an easily wearable sensor that measured 3D gait. The 3D motion analysis is considered accurate and is used as the gold standard in gait analysis [16]. Moreover, we used conceptual models to summarize various parameters of gait factors. Previous studies have also used a conceptual model [7, 8, 17–19]; however, gait assessments in all those articles were performed using a walkway (GAITRite, CIR System Inc.). The nature of walkways determined that it can only assess general (velocity, width, cadence, and stride time) and temporal (time for swing, stance, single and double support, and turning) parameters, while those used in this study could additionally evaluate gait from spatial and clearance aspects, benefit from further factors of initial contact, propulsion, and mid-swing were output, which made the gait analysis more comprehensive. Hence, compared to most other studies using simple techniques, our results are more precise and show a complete relationship between gait and cognitive function.

After adjusting for several confounders, the results showed that only general cycle factors (predominantly cadence, speed, and stance time) were significantly associated with global cognitive function in all the participants. People with lower scores for the general cycle factor were inclined to have higher MoCA-J scores. Internal biomechanism can be referred to as the proved mechanism between gait speed and cognitive function. Some lesions of the brain, such as an increased proportion of the periventricular and subcortical white matter hyperintensities, atrophy of the medial temporal areas, hippocampal atrophy [20], or small gray matter volumes in the bilateral cortical and subcortical regions, based on magnetic resonance imaging [21] can slow the gait speed, while simultaneously impairing cognitive function. In addition, recent studies have started researching the biomechanism between other parameters and cognitive function; for example, higher cerebral amyloid-β deposition was shown to be associated with increased double support time [22]. Therefore, by involving more gait aspects, a more robust mechanism could be clear between gait and cognitive function.

With respect to newly invented factors of initial contact, propulsion, and mid-swing, no significance was found for global cognitive function. Although previous studies claimed that the contributor of stride length in the initial contact factor was related to cognitive function [23], their relationship was probably caused by the high correlation with gait speed. In addition, according to a study of brain dynamics while walking, electrocortical activity progressively decreases in the pre-swing phase and acceleration phase [24], which indicates that compared to the preparatory phases for the most important point of walking, the general cycle factor that includes characteristics of posture control (double support and foot-flat) is better associated with global cognitive function.

Further exploration of cognitive domains detected by MoCA-J supported the strong association between executive function and gait [25, 26]. The term executive function refers to the higher-level cognitive skills we use to control and coordinate our other cognitive abilities and behaviors [27] traditionally, it is associated with the frontal lobes and related brain networks, in particular, the dorsolateral prefrontal cortex and cingulate cortex [28]. A previous study found that gait shared similar brain regions in which a great burden of subcortical white matter hyperintensities on magnetic resonance imaging is related to increased dual-task costs while walking [29], which could be the main reason for their high correlation. However, some individual gait parameters have a significant relationship with language, memory and attention domain [30]. After being detained by the factor analysis, combined gait characteristics did not show any relationship with other domains except for executive function. Therefore, enough evidence for an association between gait and the other two domains, orientation and visuospatial, in all the participants was not found [31].

Moreover, our results varied greatly in terms of sex. According to basic characteristics, men performed worse on both gait and cognitive assessment than women, which is proved by the previous studies that older women usually outperformed on the test of motor speed [32] and memory than men despite the same level of cognitive degeneration [33, 34]. Moreover, women showed a stronger relationship between gait and global cognitive function, and no significance relationship was observed in men. The cognitive benefit of physical activity may be greater in women than in men [35]; however, the effect and biological mechanism of sex on the relationship between gait and cognitive function remains unclear. These observations illustrate that sex difference is an important confounding factor in the study of gait and cognitive function among older adults, and it should be discussed in future work.

This study has both strengths and limitations. Although the advanced device and conceptual models used in this study makes it more comprehensive and precise, the following aspects still warrant some attention: first, this was a cross-sectional study, which meant that the causality between gait and cognitive function could not be determined. Second, during the process of measuring gait parameters, the first and last two cycles were not excluded given the short total distance, while the initiation and termination of gait are sometimes thought to be unstable and inaccurate [36]. As claimed by other studies, older adults need at least four gait cycles to reach steady-state walking speed [37]. Third, only the MoCA-J questionnaire was used in the cognitive assessment procedure, although the MoCA was identified as an applicable method for evaluating global cognition, the internal subtests of domains are not comparatively sensitive or specific for identifying attention or language impairments [38, 39]. To maintain the veracity of cognitive function, especially in the study of exploring different cognitive domains, extensive neuropsychological batteries should be added in the future research. Finally, the participants in our study were older than 70 years, and more than 70% of them had mild cognitive impairment. However, the prevalence of MCI among older adults usually ranges between 3 and 42% [40]. Atypical MCI prevalence may be associated with potential physical functional impairments and disease history of the participants, which we did not exclude before analysis, and this may make it difficult to generalize the results to a wider population.

## Conclusion

Despite an advanced gait measurement was adopted and the most various gait parameters was included in this research, significant associations were only found between general cycle and global cognitive function. In addition, this study proved the strong associations of gait with executive function. In the future, longitudinal cohort research with larger sample size and extensive neuropsychological batteries are needed to detect the causality between gait and cognitive function.

## Supplementary Information

**Additional file 1.**

## Data Availability

The datasets used and/or analyzed during the current study are available from the corresponding author on reasonable request.

## Abbreviations

CARE-DO: Cognition and Activity in Rural Environment of HokkaiDO Senior
Jages: Japan Gerontological Evaluation Study
IADL: Instrumental Activities of Daily Living
MoCA-J: Japanese version of Montreal Cognitive Assessment
MCI: Mild Cognitive Impairment
3D: Three-dimension
