# Supplementary material for: Association of gait with global cognitive function and cognitive domains detected by MoCA-J among community-dwelling older adults: a cross-sectional study
Source: BMC Geriatr. 2021 Oct 2;21:523. doi: 10.1186/s12877-021-02467-5 (PMC8487567; doi:10.1186/s12877-021-02467-5)
Supplement: Supplementary file 1 — Additional file 1. [file 12877_2021_2467_MOESM1_ESM.docx]

**Additional file 1**. Definition of each gait parameter

**Cycle duration (second)** is the duration of one cycle.

**Cadence (step/minute)** is the number of cycles in a minute.

**Stride length (meters)** describes the distance between two successive footprints on the ground, from the heel of a foot to heel of the same foot, one cycle after.

**Stride velocity (meters/s** is the forward speed of one cycle.

**Turning angle (degree)** is the angle between two consecutive foot-flat phases of the same foot on a horizontal plane.

**Stance (second)** is the time when foot is touching the ground

**Swing (second)** is the time when foot is in the air and does not touch the ground.

**Loading(second)** is the time between the heel strike and the foot being flat on the ground.

**Foot-flat(second)** is the time when foot is fully flat on the ground

**Pushing(second)** is the time when the foot being flat on the ground and the toe leaving the ground at take-off

**Double support(second)** is the time when both feet touch ground

**Peak angle velocity(degree/s)** is the maximum angular velocity during the swing phase, between max heel clearance and min toe clearance

**Swing speed(meters/s)** is the maximum forward speed of the foot during swing

**Strike angle (degree)** is the angle between the foot and the ground at heel strike, on a vertical plane.

**Lift-off angle (degree)** is the angle of the foot at the end of the push phase, just at take-off

**Swing width (meters)** is the maximum lateral excursion of the foot during swing phase

**3d path length (meters)** is the length of real path of the foot during one cycle in 3d space, including both stride length and swing width.

**Max. Heel Clearance (meters)** is the maximal height above the ground reached by the heel during each cycle.

**Max. Toe Clearance 1 (meters)** is the maximal height above the ground reached by the toes just after heel max clearance

**Min. Toe Clearance (meters)** is the minimum height of the toes during swing phase.

**Max. Toe Clearance 2 (meters)** is the maximal height above the ground reached by the toes just before heel strike.

| **Additional table 2**. Adjusted means of internal subtests by tertile of each gait factor in women (N=111) | | | | | | | |
| --- | --- | --- | --- | --- | --- | --- | --- |
| **Factor** |  | **Executive Function** | **Language** | **Memory** | **Orientation** | **Visuospatial** | **Attention** |
|  |  |  |  |  |  |  |  |
| General cycle | low | 2.88 | 3.68 | 3.30 | 5.79 | 2.67 | 5.02 |
|  | moderate | 2.75 | 3.72 | 3.13 | 5.92 | 2.73 | 4.96 |
|  | high | 2.42 | 3.72 | 2.80 | 5.53 | 2.41 | 4.53 |
| *P* for trend |  | 0.208 | 0.749 | 0.228 | 0.085 | 0.482 | 0.172 |
|  |  |  |  |  |  |  |  |
| Initial contact | low | 2.38 | 3.76 | 2.95 | 5.82 | 2.62 | 4.84 |
|  | moderate | 2.85 | 3.77 | 3.41 | 5.78 | 2.68 | 5.00 |
|  | high | 3.21 | 3.49 | 3.03 | 5.64 | 2.53 | 4.80 |
| *P* for trend |  | 0.007 | 0.134 | 0.903 | 0.413 | 0.281 | 0.510 |
|  |  |  |  |  |  |  |  |
| Propulsion | low | 2.94 | 3.84 | 3.18 | 5.89 | 2.63 | 5.01 |
|  | moderate | 2.60 | 3.78 | 3.22 | 5.86 | 2.61 | 4.68 |
|  | high | 2.71 | 3.59 | 3.04 | 5.65 | 2.62 | 4.95 |
| *P* for trend |  | 0.428 | 0.238 | 0.717 | 0.090 | 0.730 | 0.836 |
|  |  |  |  |  |  |  |  |
| Mid-swing | low | 2.64 | 3.70 | 3.17 | 5.64 | 2.50 | 4.81 |
|  | moderate | 2.91 | 3.63 | 3.26 | 5.91 | 2.73 | 5.03 |
|  | high | 2.68 | 3.86 | 2.72 | 5.91 | 2.82 | 4.85 |
| *P* for trend |  | 0.923 | 0.770 | 0.616 | 0.023 | 0.319 | 0.984 |
| All scores are displayed as least-squares mean values adjusted for age, education, and height; *P* for trends were tested by linear regression models adjusted for age, education, height, weight, diabetes, hypertension, and IADL scores. | | | | | | | |

| **Additional table 3**. Adjusted means of internal subtests by tertile of each gait factor in men (N=135) | | | | | | | |
| --- | --- | --- | --- | --- | --- | --- | --- |
| **Factor** |  | **Executive Function** | **Language** | **Memory** | **Orientation** | **Visuospatial** | **Attention** |
|  |  |  |  |  |  |  |  |
| General cycle | low | 2.92 | 3.69 | 2.72 | 5.72 | 2.39 | 5.20 |
|  | moderate | 2.75 | 3.85 | 2.41 | 5.73 | 2.65 | 4.90 |
|  | high | 2.65 | 3.86 | 2.31 | 5.67 | 2.68 | 5.10 |
| *P* for trend |  | 0.301 | 0.284 | 0.362 | 0.802 | 0.096 | 0.995 |
|  |  |  |  |  |  |  |  |
| Initial contact | low | 2.77 | 3.90 | 2.52 | 5.62 | 2.54 | 5.02 |
|  | moderate | 2.63 | 3.78 | 2.25 | 5.65 | 2.59 | 4.89 |
|  | high | 2.82 | 3.82 | 2.52 | 5.79 | 2.66 | 5.19 |
| *P* for trend |  | 0.705 | 0.815 | 0.742 | 0.259 | 0.444 | 0.300 |
|  |  |  |  |  |  |  |  |
| Propulsion | low | 2.74 | 3.65 | 2.46 | 5.61 | 2.59 | 5.11 |
|  | moderate | 2.68 | 4.02 | 2.21 | 5.81 | 2.65 | 5.14 |
|  | high | 2.88 | 3.85 | 2.77 | 5.71 | 2.58 | 4.75 |
| *P* for trend |  | 0.769 | 0.146 | 0.701 | 0.373 | 0.950 | 0.354 |
|  |  |  |  |  |  |  |  |
| Mid-swing | low | 2.42 | 3.65 | 2.56 | 5.67 | 2.71 | 5.00 |
|  | moderate | 2.80 | 4.05 | 2.18 | 5.71 | 2.75 | 5.07 |
|  | high | 2.81 | 3.72 | 2.57 | 5.71 | 2.47 | 5.05 |
| *P* for trend |  | 0.190 | 0.367 | 0.715 | 0.920 | 0.052 | 0.755 |
| All scores are displayed as least-squares mean values adjusted for age, education, and height; *P* for trends were tested by linear regression models adjusted for age, education, height, weight, diabetes, hypertension, and IADL scores. | | | | | | | |
